# The increasing incidence of surgically treated quadriceps tendon ruptures

**DOI:** 10.1007/s00167-019-05453-y

**Published:** 2019-03-27

**Authors:** Aleksi Reito, Juha Paloneva, Ville M. Mattila, Antti P. Launonen

**Affiliations:** 1 Central Finland Central Hospital, Keskussairaalantie 19, 40620 Jyvaskyla, Finland; 2 Institute of Clinical Medicine, University of Eastern Finland, Yliopistonranta 1, 70210 Kuopio, Finland; 3 Department of Orthopaedics, Unit of Musculoskeletal Surgery, Tampere University Hospital, Teiskontie 35, 33521 Tampere, Finland; 4 School of Medicine, University of Tampere, 33014 Tampere, Finland

**Keywords:** Quadriceps tendon, Tendon rupture, Registry study, Epidemiology, Incidence

## Abstract

**Purpose:**

Due to increased life expectancy and a more active life style of the older people, we hypothesised that the incidence of quadriceps tendon rupture (QTR) is higher than previously reported. The objective of this nationwide population-based study was to investigate the annual incidence of QTR in patients who underwent operative treatment in Finland between 1997 and 2014.

**Methods:**

The Finnish National Hospital Discharge Register was searched for all adult patients who had undergone surgical treatment for QTR during the study period. Population-based annual incidence and incidence trends for surgically treated QTR were calculated.

**Results:**

During the study period, 1343 QTR surgeries were performed. Of these, 90% were performed on male patients. The incidence of QTR increased by 411% from 0.55 to 2.82 per 100,000 person-years from 1997 to 2014. The average yearly increase in the number of surgeries was 9.0%. In male patients, the incidence of QTR increased by 490% and in female patients by 121%. The mean age of the male patients increased during the study period from 50 to 59 years.

**Conclusions:**

Based on the data from the Finnish National Hospital Discharge Register, the annual incidence of QTR increased by over 400% during the 18-year study period. The average age of the patients also increased. Because QTR is only very rarely treated without surgery, the results of our study can be considered to be a reliable estimate of the increase in the incidence of this condition. There is an urgent need to establish the risk factors associated with QTR and to also establish the optimal surgical technique.

**Level of evidence:**

IV.

**Electronic supplementary material:**

The online version of this article (10.1007/s00167-019-05453-y) contains supplementary material, which is available to authorized users.

## Introduction

Along with other soft-tissue injuries that affect elderly patients, such as Achilles tendon rupture (ATR), patellar tendon rupture (PTR), rotator cuff (RC) lesions, and biceps tendon rupture (BTR), quadriceps tendon rupture (QTR) can also be seen as an end stage of chronic tendon degeneration and overuse [15, 24]. Moreover, knee extensor mechanism injuries may also result from quadriceps or patellar tendon rupture or patellar fractures (PF) [9, 12, 23]. More than half of such injuries are PFs, and they usually occur in older female patients [9, 23]. Traditionally, QTR has been regarded as a relatively rare soft-tissue injury that affects elderly patients [6].

The mean age of patients suffering a QTR has been reported to vary between 47 and 69 years [5, 9, 15], and QTR is most commonly seen after a simple fall [5, 9, 12, 23]. The rupture commonly follows a sudden contracture of the quadriceps muscle, although spontaneous ruptures do occur [12]. Contrary to other tendon ruptures that affect older patients, non-operative treatment is very seldom reasonable option for QTR [5]. Numerous surgical methods for the treatment of QTR have been proposed, but pooled analyses of level IV studies have found no differences between the different methods with regards to clinical outcomes, complications, and adverse events [5, 21]. Moreover, even after immediate surgical repair, muscle atrophy, strength deficit, and patellofemoral joint incongruency are commonly seen [5].

At present, there is a scarcity of literature on the incidence of QTR. Only one study reporting the incidence of QTR has been published. In this report from the UK, the annual incidence was 1.37 patients per 100,000 persons [6]. Furthermore, a growing body of research indicates that the incidence of other degenerative tendon ruptures is also increasing. For example, the incidence of ATR has increased in recent years [8, 10, 11, 16], and this increase is especially prevalent in older patients [8, 11]. In addition, recent studies suggest that the incidences of distal BTRs and RC lesions are also increasing [13, 17, 19, 20]. The major etiological factors that have been suggested to explain these changes are an increase in life expectancy and a more active life style [1, 3]. Together, these factors have resulted in a higher incidence of musculoskeletal degeneration-related diseases. Therefore, it is reasonable to assume that the incidence of QTR has also undergone a similar rising trend.

Accurate morbidity figures caused by degenerative tendon ruptures are urgently needed. The previous studies reporting the increasing incidence of degeneration-related tendon injuries and lesions have called for the application of preventive measures [8, 17]. Incidence of QTR is largely unknown. QTR is a serious injury and accurate epidemiological data are needed to allocate treatment resources. Sharing these perspectives, the objective of this nationwide population-based study was to investigate the annual incidence of QTR in patients who underwent operative treatment in Finland during the years 1997–2014.

## Materials and methods

The Finnish National Hospital Discharge Register (NHDR) provides data on the age, sex, domicile of the subject, length of stay, primary and secondary diagnoses, and associated surgical procedures for all in-patient and hospital visits in Finland. Data collection by the NHDR is mandatory for all public and private hospitals and other institutions providing any kind of care in Finland.

The NHDR database was searched for all patients who had undergone a surgical procedure for a ICD-10 code S76.1 *Injury of quadriceps muscle and tendon*. From these patients, all patients with a procedural code of NFL30 *Repair or transposition of muscle of hip or thigh*, according to the Nordic Classification of Surgical Procedures (NCSP) were included. All duplicate patients were excluded from the data, and only the first instance of surgery was included. Repairs of the patellar tendon are not assigned to these codes, since they have their unique codes, namely NGL30 *Repair or re-insertion of the patellar tendon*. All adult patients aged 18 years or over who had been discharged between 1997 and 2014 were included. Ethical approval for this study was granted by the Finnish National Institute of Health and Wellness (Dnr THL/89/5.05.00/2012, dated January 18, 2012).

### Statistical analysis

To calculate the incidence rate of quadriceps tendon repairs, the annual adult population living in Finland was obtained from the online registry maintained by Official Statistics of Finland. The registry provides data on the size of the population on the last day of each study year. During the study period, the total adult population at risk of quadriceps tendon injury increased by 10.2% from 3.989 million in 1997 to 4.396 million in 2014. The annual incidence of quadriceps tendon repairs per 100,000 persons was calculated. Since the number is based on the entire population of Finland, 95% confidence intervals (CIs) were not calculated. The average change in the number of surgically treated QTRs was estimated using both negative binomial and Poisson regression. In this way, it was aimed to assess any possible overdispersion. The total number of annual operations was used as a dependent variable and year as an independent variable. In these models, the regression coefficient *β*, for year, estimates the average proportional change per year. Then, 95% CIs were calculated for the coefficients. All statistical analyses were performed with R software v3.2.4 (R Foundation for Statistical Computing, Vienna, Austria). Since this study was based on a national register, no sample size calculation was performed.

## Results

In total, 1343 QTR surgeries were performed on 1343 patients during the study period. Of these, 1202 (89.7%) of the operations were performed on male patients. During the whole-study period, the mean age of males was 55 (SD 13) and the mean age of females was 59 (SD 16) years.

The incidence of QTR increased by 411% from 0.55 to 2.82 per 100,000 person-years from 1997 to 2014. The annual number of surgeries varied from 22 to 161 (median 67.5). The average annual increase in the number of surgically treated QTRs was 9.0% (95% CI 7.9–10.1) using Poisson regression. This was similar to that seen in negative binomial regression [8.9% (95% CI 7.0–10.8)].

**Fig. 1:**
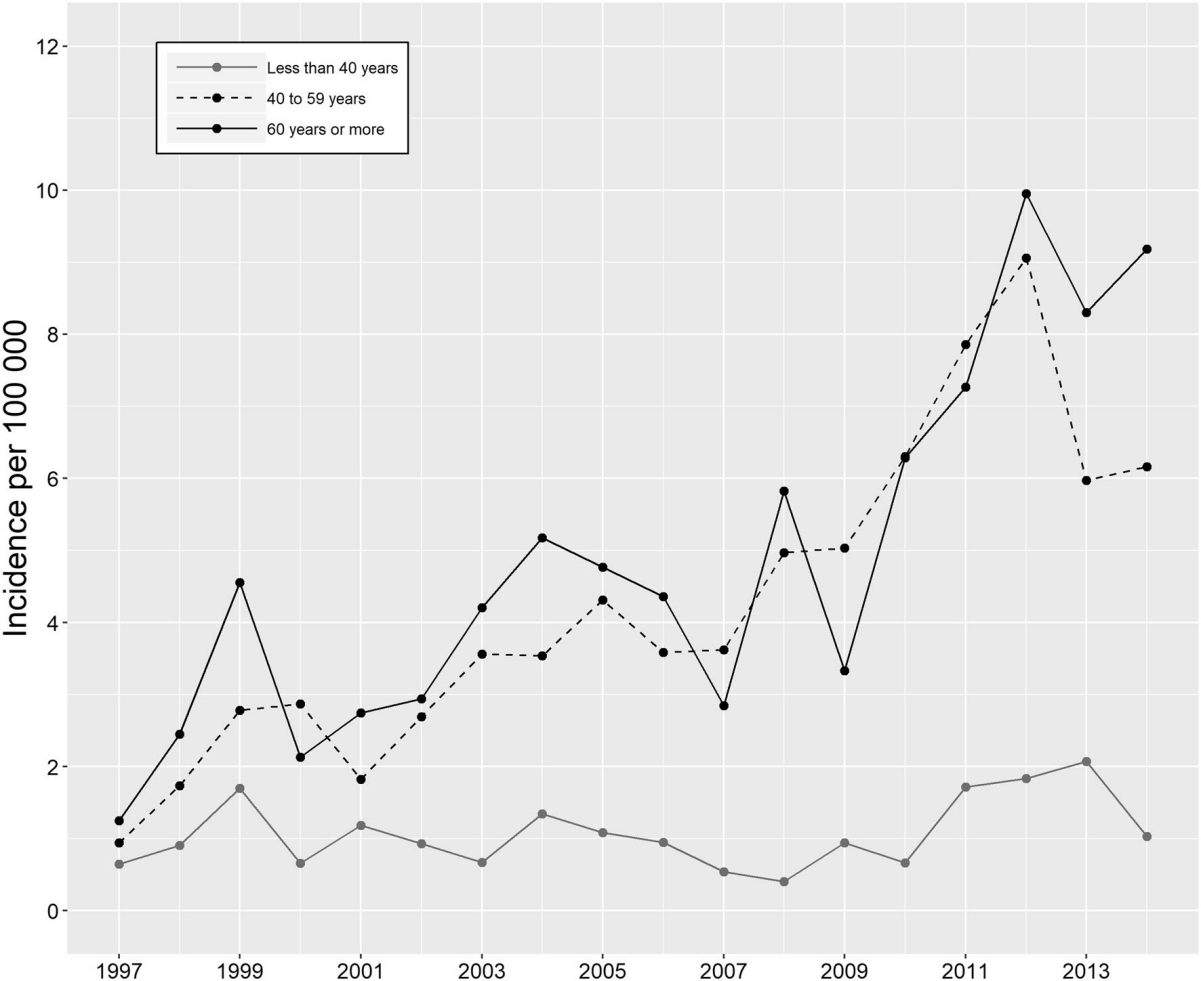
Annual incidence of operatively treated QTRs in male patients

In male patients, the incidence of QTR increased by 490% from 0.89 to 5.23 per 100,000 person-years from 1997 to 2014 (Supplementary Table 1). The increase was most profound in patients aged from 40 to 59 years and 60 years or more. In patients aged under 40 years, the increase was very subtle (Fig. 1).

**Fig. 2:**
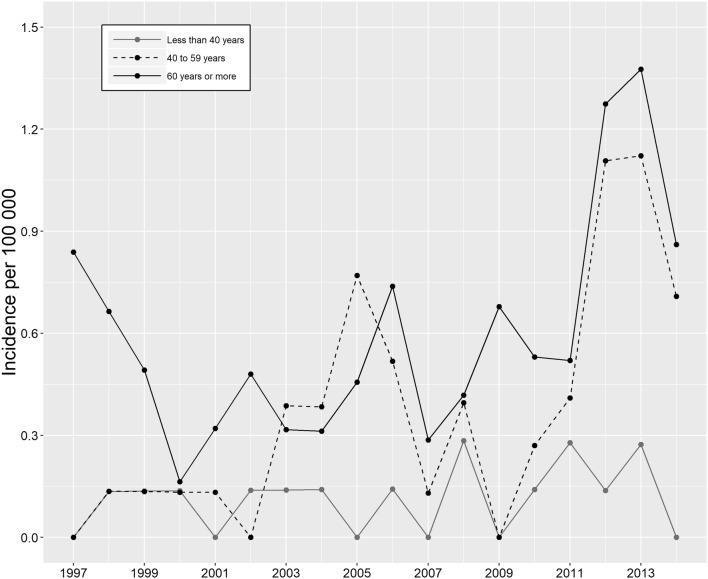
Annual incidence of operatively treated QTRs in female patients

In female patients, the incidence increased by 121% from 0.24 to 0.53 per 100,000 person-years from 1997 to 2014 (Supplementary Table 1). As was the case in male patients, the change was the most notable in the older age groups, and the increase was subtle or negligible in the younger age group (Fig. 2).

The mean age of the male patients increased from 49.5 years in 1997 to 58.8 years in 2014 (Supplementary Table 1). In female patients, the corresponding numbers were 67.2 years in 1997 and 60.9 years in 2014, showing no clear temporal trend (Supplement).

## Discussion

The most important finding of the present study was the steep increase in the annual incidence of operatively treated QTRs from 1997 to 2014. To the best of our knowledge, this is the first study to assess the nationwide incidence of surgically treated QTRs. During the 18-year study period, there was a 4.1-fold overall increase in the incidence of QTRs. Along with the increasing incidence of QTRs, especially in male patients, it was also evident that the mean age of the patients suffering this injury increased, because the increase in incidence was the most dramatic in patients aged 60 or more.

Huttunen et al. [11] reported that the overall incidence of ATRs in Sweden has risen steadily in recent years, and that there has also been a clear increase in the mean age of the patients suffering from the condition. One likely explanation for the increase in the mean age of patients sustaining an ATR is the growing number of older patients participating in high-demand sports [1, 3]. The more active life style of older patients may have also contributed to the findings in our study. In our study, the mean age of the male patients sustaining a QTR increased from 49.5 to 55.8 years during the 18-year study period. It is crucial to note, however, that QTR is rarely directly associated with participation in high-demand sports and is more often associated with a degenerative tendon or a simple fall [5, 9]. Naturally, a fall that results in QTR could very well happen during high-demand sports also. The aetiology of QTR could not, however, be evaluated from the available register data.

It can be postulated that a more active lifestyle plays a less important role in the changing epidemiology of QTR compared with that of ATR. As previously mentioned, QTR usually occurs after a simple fall and a sudden uncontrolled muscle contracture that results in a rupture in a degenerated tendon [15, 24]. Tendon degeneration is correlated with chronic illnesses, such as diabetes, hyperlipidemia, and thyroid disorder [2, 9, 14]. In a large series of patients with a knee extensor mechanism injury, Garner et al. reported that patients with QTR were more obese than those with a PF, which also commonly follows a simple fall [9]. The incidence of chronic diseases and obesity associated with QTR and PF has been steadily increasing at a national level [22]. Therefore, it is likely that, together with an increasing overall life expectancy, older patients are more vulnerable to conditions associated with degeneration, such as QTR.

The main limitation in our study was the lack of information regarding the possibility that the patient had undergone a total knee arthroplasty (TKA). QTR is a rare although devastating complication after TKA that occurs most commonly during the first months after surgery [4, 7]. Whereas spontaneous QTR is usually an endpoint in the tendon degeneration process, the predisposing factors after TKA include a possible iatrogenic injury to the tendon during surgery. Therefore, these two rupture types must be considered as different entities. The prevalence of QTR rupture in patients having undergone a TKA has ranged between 0.1 and 0.29% in two large cohort studies [4, 7]. During our study, the number of annual TKAs performed in Finland increased from 4279 in 1997 to 10,406 in 2014 [18]. Since 2006, the increase has plateaued with the number of annual TKAs varying between 9541 and 10,917. Assuming that the prevalence of QTR during the early postoperative period after TKA was as high as 0.29% in the Finnish population, the annual number of QTRs associated with TKA would range from 27 to 31 patients and would, therefore, not explain the increase seen in our study. Although the number of TKAs performed annually had possibly doubled during the study period, the extent of the contribution of QTR associated with TKA in the observed increase in the incidence of QTRs was small. The majority of the observed three-to-fivefold increase in incidence is, therefore, likely due to other factors.

Evidence for the optimal surgical treatment of patients sustaining a QTR is very limited. No randomised clinical trials (RCT) that have compared different surgical treatment methods have been published. An important part of treatment is also the postoperative regime, which can vary in both time and immobilisation method used. There is no evidence of the optimal postoperative treatment regime either. It is feasible and intuitive to choose the method of surgical repair according to the site of the tear. Paradoxically, while the incidence of QTR has risen remarkably, RCTs that compare different postoperative treatments are lacking. The patients sustaining a QTR are older than patients with other extensor mechanism injuries, such as patellar fracture or PTR. Therefore, these patients are more vulnerable to immobilisation-related comorbidities, such as venous thromboembolism and re-ruptures [21]. Moreover, due to their advanced age, these patients are also more prone to heterotopic ossification and deep infection, which may have devastating outcomes and long-term morbidity [21].

A further limitation in this study was the lack of complete data on comorbidities. Since these are incompletely recorded in the database, it was not possible to investigate the effects of comorbidities on the incidence of QTRs, of which diabetes and endocrine diseases would have been of most interest. The major strength of our study was the inclusion of nationwide data that minimised bias and increased robustness in the analysis leaving only coding errors as a main source of uncertainty and bias.

Based on data from the Finnish National Hospital Discharge Register, the annual incidence of QTR increased by 411% during the 18-year study period. The average age of the patients affected by the injury also increased. In total, 9 out of 10 QTRs occurred in male patients. QTR is very rarely treated without surgery, and thus, our study result can be considered to be a reliable estimate of the increase in the incidence of this injury.

## Conclusion

The incidence of QTR is higher than previously reported, and it has increased steeply in recent years. Moreover, this injury is usually sustained by patients aged 50 years or more who may be more prone to comorbidities associated with surgery. Our study adds to the growing body of evidence that the increasing incidence of degenerative tendon injuries and lesions, especially in older male patients, calls for the need for preventative measures. Following the steep increase in the incidence of QTR and the larger volume of patients sustaining this injury, there is also an urgent need to establish the risk factors associated with QTR and to establish the optimal surgical treatment regime.

## Electronic supplementary material

Below is the link to the electronic supplementary material.

Supplementary material 1 (DOCX 15 KB)

## Abbreviations

QTR: Quadriceps tendon rupture
ATR: Achilles tendon rupture
PTR: Patellar tendon rupture
NHDR: National Hospital Discharge Register
SD: Standard deviation
TKA: Total knee arthroplasty
