# Supplementary material for: The increasing incidence of surgically treated quadriceps tendon ruptures
Source: Knee Surg Sports Traumatol Arthrosc. 2019 Mar 27;27(11):3644–9. doi: 10.1007/s00167-019-05453-y (PMC6800863; doi:10.1007/s00167-019-05453-y)
Supplement: Supplementary file 1 — Supplementary material 1 (DOCX 15 KB) [file 167_2019_5453_MOESM1_ESM.docx]

**Table 1**: Annual incidence of QTR repairs and the mean age of the patients throughout the study years divided by gender.

|  | **Males** | | **Females** | |
| --- | --- | --- | --- | --- |
| Year | Incidence per 100 000 | Mean age | Incidence per 100 000 | Mean age |
| 1997 | 0.89 | 49.5 | 0.24 | 67.2 |
| 1998 | 1.56 | 52.5 | 0.29 | 69.3 |
| 1999 | 2.73 | 51.6 | 0.24 | 55.4 |
| 2000 | 1.85 | 51.4 | 0.14 | 45.7 |
| 2001 | 1.78 | 50.6 | 0.14 | 61.0 |
| 2002 | 2.08 | 53.1 | 0.19 | 59.3 |
| 2003 | 2.62 | 54.5 | 0.28 | 53.2 |
| 2004 | 3.10 | 53.7 | 0.28 | 58.5 |
| 2005 | 3.24 | 55.1 | 0.42 | 56.4 |
| 2006 | 2.82 | 56.5 | 0.46 | 55.8 |
| 2007 | 2.31 | 54.6 | 0.14 | 67.3 |
| 2008 | 3.55 | 58.2 | 0.37 | 54.5 |
| 2009 | 3.09 | 55.1 | 0.23 | 73.6 |
| 2010 | 4.27 | 57.0 | 0.32 | 60.0 |
| 2011 | 5.48 | 54.8 | 0.41 | 54.2 |
| 2012 | 6.71 | 56.8 | 0.85 | 61.7 |
| 2013 | 5.26 | 56.9 | 0.94 | 58.3 |
| 2014 | 5.23 | 58.8 | 0.53 | 60.9 |
